# Emerging Issues in Occupational Safety and Health

**DOI:** 10.3390/ijerph15122897

**Published:** 2018-12-18

**Authors:** Kapo Wong, Alan Hoi Shou Chan

**Affiliations:** Department of Systems Engineering and Engineering Management, City University of Hong Kong, Tat Chee Avenue, Kowloon, Hong Kong, China; kpwong42-c@my.cityu.edu.hk

Working environments have various risks, which result in accidents and casualties. To prevent and minimize the occurrence of avoidable accidents, all workers should understand occupational safety and health. The principle of occupational safety and health is to safeguard the safety and health of workers through the establishment of a safe working environment. The protection of occupational safety and health involves multitudinous categories, such as ergonomics, toxicology, physics, chemistry, and economics. The concern for the occupational safety and health of workers focuses not only on their physical health but also on mental health. Mental health refers to the well-being of an individual. Poor mental health may be a predictor of physical health problems and people with physical illnesses have a high tendency of suffering from depression and distress [1]. However, according to a survey conducted by *Safety and Health Magazine* in 2018, about 50% of the respondents thought that mental health should not be counted as part of occupational safety and health [2]. In fact, mental health is part and parcel of occupational safety and health [3,4]. No matter what types of occupational diseases and illnesses workers experience, deleterious effects will be generated, for example, low productivity [5], high rates of absenteeism [6], and economic loss for companies [7]. Furthermore, dynamic and changing working environments result in many unknown risks, which pose challenges and opportunities for workers, organisations, and authorities. Therefore, potential risks in working environments should be eliminated by identifying factors affecting risk-taking behaviour and the mental and physical health of workers, pertinent health measures, approaches on occupational safety and health in organisations, and government legislation. The published papers in the Special Issue on “Emerging Issues in Occupational Safety and Health” cover all these important and eminent issues.

To identify factors affecting the risk-taking behaviour of workers, several studies have investigated the reasons why they conduct such behaviour [8,9,10,11,12,13]. Factors influencing the risk-taking propensity of workers include safety supervision and inspection, safety culture of working environments, social influence, workplace conditions, attitude of workers towards risk, risk perception, and self-perceived easiness of risk-taking behaviour [8,9]. Jiang and Han [10] report that the preconditions for unsafe acts, unsafe supervision, and organisational influences resulted in unsafe behaviour. Larsson et al. [11] demonstrate that the safety perception of workers was influenced by safe working environments, leadership, prioritising safety at work, and provision of trust, and support by management. Tong et al. [12] explore human risk factors affecting risk-taking behaviour through coal mining roof accidents in China; such factors include knowledge, information, communication, and performance and behaviour of senior managers. Thamrin et al. [13] identify inadequate safety training, long working hours (more than 20 h per week), and insufficient confidence on the discussion of safety issues as the main factors resulting in injuries. Therefore, the awareness and consciousness on occupational safety and health should be enhanced by improving these identified areas to avoid performing unsafe behaviour that will adversely affect the physical and mental health of workers.

The physical and mental health problems of workers can tremendously affect their work performance. Numerous factors can lead to physical and psychological occupational diseases and illnesses [14,15,16,17,18,19,20,21,22]. Kim and Cho [19] find that Korean workers with a high demand on childcare easily contracted musculoskeletal disorders and had problems with work–life conflicts. High concentrations of asbestos in factories threaten health of workers and cause a number of deaths and disabilities every year [17]. Molino et al. [22] point out a negative relationship between exhaustion and recovery among workaholism. According to a cluster analysis of high-frequency words with long working hours as the core theme [20], physiological health problems are not the main aspect that causes the burnout of workers, and psychological distress has gradually become the primary concern. Job stress is one of the prevailing psychological diseases and negatively influences the performance of workers [14]. Junne et al. [16] also state that stress is a “pandemic” disease among workers in Germany and suggest that different sectors should cooperate to improve the mental health of workers. However, Ma et al. [21] note that an appropriate amount of challenge stress should be put onto workers to ameliorate worker quality. Furthermore, female workers experience more health problems and lower psychological well-being than male workers [15]. Female workers also have a higher incidence of symptomatic cervical and lumbar disc herniation compared with male workers [18]. All these studies in this Special Issue reveal that the influences of physical and mental health problems cannot be neglected; thus, measures should be developed for evaluation of the health status and work performance of workers to reduce unnecessary accidents and risks.

Sensitive and quick measures should be developed to effectively assess the health condition of workers and minimize the severity level of certain occupational health problems. Fatigue is one of the hidden syndromes of many diseases. Duan et al. [23] introduced a mental fatigue detection index to alert workers of whether they suffer from chronic exhaustion and propose countermeasures to alleviate this problem. Job stress is a concerned psychological health problem among most workers. To measure the job stress of workers, Wu et al. [24] developed a job stress scale for construction workers in China given that job stress causes different types of unsafe behaviour in the construction industry. A number of work performance measures were also developed to appraise the capability of workers. Job satisfaction measurements were proposed to identify several facet items, such as stress and work engagement [25,26]. The Work Ability Index was used to predict the work performance of workers for work design and improvement [27]. These measures may be able to resolve the instant health problems of workers while appropriate strategies and policies should be formulated to address related health issues.

Other than the efforts of workers and researchers, the work organisation should establish a positive, safe culture in the working environment. Wagner et al. [28] suggest that the existence of worker safety culture is not sufficient and patient safety culture should be incorporated to develop a safe working environment in hospitals. Governments and organisations should offer continuing education and training for workers to deepen their consciousness on occupational safety and health and provide compensation for victims [14,29]. Legislation and guidance protocols should be implemented to address the safety and health issues of workers [30]. Undeniably, a considerable amount of direct costs and indirect costs are involved for all of these actions [7,31]. However, in the long run, the safety and health of workers can be safeguarded, thereby reducing absenteeism and turnover rates, increasing the attraction of talent and goodwill of companies, enhancing worker motivation and organisation commitment, and reducing costs on healthcare and social insurance.
